# Role of the ESAT-6 secretion system in virulence of the emerging community-associated *Staphylococcus aureus* lineage ST398

**DOI:** 10.1038/srep25163

**Published:** 2016-04-26

**Authors:** Yanan Wang, Mo Hu, Qian Liu, Juanxiu Qin, Yingxin Dai, Lei He, Tianming Li, Bing Zheng, Fan Zhou, Kaiwen Yu, Jingyuan Fang, Xiaoyun Liu, Michael Otto, Min Li

**Affiliations:** 1 Department of Laboratory Medicine, Renji Hospital, School of Medicine, Shanghai Jiaotong University, Shanghai 200127, China; 2 Institute of Analytical Chemistry and Synthetic and Functional Biomolecules Center, College of Chemistry and Molecular Engineering, Peking University, Beijing 100871, China; 3 Division of Gastroenterology & Hepatology, Renji Hospital, School of Medicine, Shanghai Jiaotong University; Shanghai Institute of Digestive Disease, Shanghai 200127, China; 4 Pathogen Molecular Genetics Section, Laboratory of Bacteriology, National Institute of Allergy and Infectious Diseases, The National Institutes of Health, Bethesda, MD 20892, USA

## Abstract

Novel *Staphylococcus aureus* clones continue to emerge that cause infections in otherwise healthy people. One example is the sequence type (ST) 398 lineage, which we show here is increasing in importance as a significant cause of community-associated (CA) human infections in China. We have a profound lack of understanding about what determines the considerable virulence potential of such newly emerging clones. Information about the contribution to virulence of the more recently discovered ESAT-6 secretion system (ESS) has remained particularly scarce. The Chinese ST398 isolates exhibited significantly increased expression of ESS genes as compared to predominant hospital-associated clones, which we found is likely due to increased expression of the accessory gene regulator (Agr) system and control of ESS by Agr. Importantly, deletion of *essB* in ST398 resulted in significantly reduced resistance to neutrophil killing and decreased virulence in murine skin and blood infection models. Our results demonstrate a key function of ESS in promoting virulence and mechanisms of resistance to innate host defense in an important emerging CA-*S. aureus* lineage. They suggest that ESS has a so far underestimated role in promoting aggressive virulence and epidemiological success of *S. aureus*.

*Staphylococcus aureus* is an important pathogen that causes a variety of infections, ranging from localized skin and soft-tissue infections (SSTIs) to life-threatening severe infections such as necrotizing pneumonia or sepsis^1^. There are reports indicating that hospital-associated infections due to methicillin-resistant *S. aureus* (MRSA) are decreasing, at least in the U.S. and Western Europe^2^,^3^. On the other hand, the considerable morbidity and mortality that is due to community-associated infections, caused by both methicillin-resistant and methicillin-sensitive *S. aureus* isolates, is increasing on a worldwide scale^2^,^4^,^5^,^6^.

By contrast with hospital-associated *S. aureus* (HA-SA) infections, for which there are predisposing risk factors, community-associated *S. aureus* (CA-SA) infections can occur in otherwise healthy individuals, suggesting that these bacterial strains have greater virulence than HA-SA strains^7^. In China, HA-SA has been extensively studied during the past years with ST239 and ST5 MRSA as the predominant clones. In comparison, data on CA-SA in China are still limited, but studies in children suggest predominance of isolates belonging to sequence type (ST) 59^8^.

The ST398 lineage was originally described in livestock and sporadically causes infections in humans working in close proximity to animals^9^,^10^. However, in the Americas, it is now also recognized as a causative agent of infections in humans living in animal-free environments^11^. Whole genome sequencing of human CA-SA ST398 revealed that it represents a human-adapted lineage that is different in genetic composition from animal-associated ST398 clones^11^. The molecular processes underlying the capacity of CA-SA ST398 to infect healthy individuals and spread sustainably in the population are poorly understood, but recent results obtained with ST398 isolates from Brazil that caused fatal pneumonia indicate an important role of Agr-controlled toxins, in particular α-toxin, in ST398 virulence^12^.

The ESAT-6 secretion system (ESS), originally described in *Mycobacterium tuberculosis*, has recently been discovered in *S. aureus*^13^. While there are strain-dependent differences, the system includes as core genes the *esaA*, *esaB*, *essA*, *essB*, *essC*, *esxA* and *esxB* genes^14^. The Ess proteins are membrane-located components of the secretion machinery, EsaB is a regulator, and the EsxA, EsxB, and EsaC are exported effector proteins^13^,^14^,^15^. Mutants in *esxA*, e*sxB*, and *essC* have been shown to contribute to abscess formation in *S. aureus* Newman^13^. Furthermore, the ESS has also been shown to impact nasal colonization and pneumonia, albeit in a strain-dependent fashion^14^. The mechanistic function in pathogenesis of secreted ESS substrates is poorly understood, but results from a recent study indicate a role of EsxA and EsxB in the modulation of apoptosis and release of ingested *S. aureus* from epithelial cells^16^.

In the present study, we show that there is a considerable increase of CA-SA infections in China that are due to ST398 and demonstrate increased virulence of CA-SA ST398 as compared to predominant HA-SA strains. We then sought to determine yet unknown factors that contribute to the enhanced virulence potential of CA-SA ST398 and discovered increased expression of ESS factors in ST398 isolates, pointing to a potential key role of ESS in ST398 virulence. By construction and analysis of an *essB* deletion mutant in ST398, we demonstrate a significant role of ESS in acute ST398 infection, which our results indicate may be due to a previously unrecognized role of ESS in the evasion of destruction by human neutrophils.

## Results

### Prevalence of CA-SA ST398 is increasing in China

**Figure 1:**
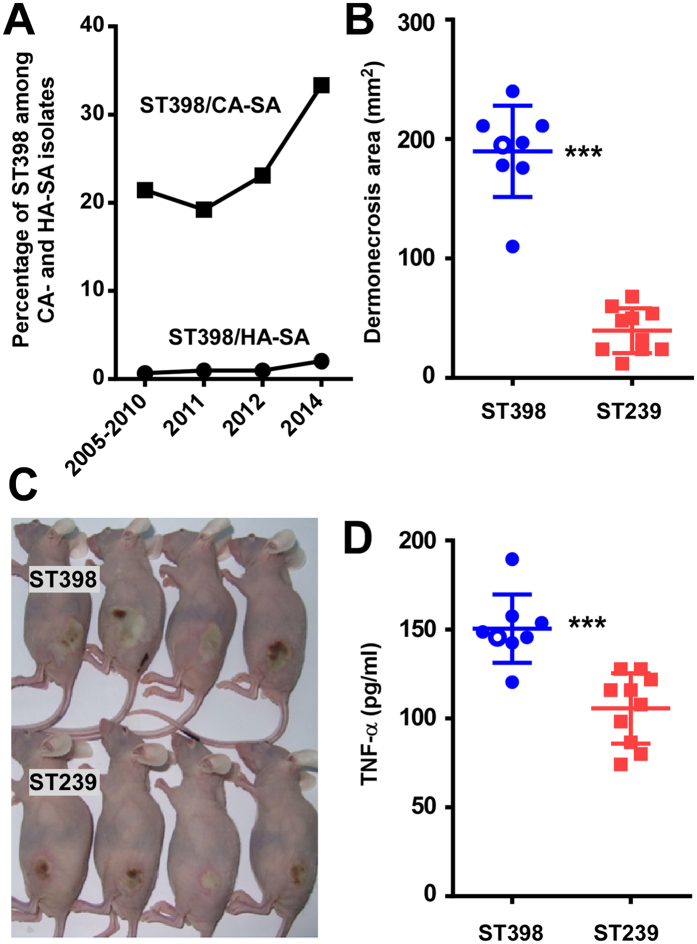
Emergence of highly virulent ST398 as a cause of CA-SA infections in China. (**A**) Percentage of CA- and HA-SA infections caused by ST398 among adult patients at Renji hospital, Shanghai. See Table 1 for absolute numbers. (**B,C**) Mouse abscess model with randomly selected isolates of CA-SA ST398 and HA-SA ST239 obtained from the same hospital and during the same time frame. Analyzed were n = 8 mice/group. Abscess development was measured at day 2 after infection. (**C**) Representative abscesses. (**D**) Concentration of TNF-α in the blood of animals at the time of euthanasia. (**B,D**) ***p < 0.001 (unpaired t-test). The empty circle represents the data corresponding to the isolate selected for genetic deletion.

Laboratory-based surveillance in Shanghai teaching hospitals indicated a considerable number of CA infections that were due to ST398 isolates, with the number of cases increasing over time and reaching ~1/3 of all CA infections in 2014 (Fig. 1A, Table 1). During the entire surveyed time frame (2005–2014), the corresponding numbers for HA ST398 cases were extremely low (overall ~1%), suggesting a community origin of the surge in ST398 cases. Data from a pediatric hospital obtained in 2012 painted a similar picture (Table 1). The majority of the CA-SA ST398 isolates belonged to spa types t034 and t571. They were mostly associated with skin, blood, and lung infections (Table 1). The *lukSF* genes encoding the Panton-Valentine leukocidin (PVL), a leukocidin often epidemiologically associated with CA-MRSA infections^17^, were present in only 27.5% of CA-SA ST398 isolates from adults and absent from pediatric isolates. The vast majority of CA-SA ST398 isolates (90%) were methicillin-sensitive. Finally, of the patients that could be reached to provide the respective information (42/69), only 3 (7%) reported contact with livestock within the last month, indicating that the surge in ST398 infections in Shanghai did not originate from livestock.

### CA-ST398 is more virulent than HA-ST239

SSTIs are the most frequent type of infections caused by CA-SA^6^. Therefore, we used a mouse abscess model to compare the virulence of randomly selected clinical CA-SA ST398 with that of randomly selected isolates of ST239, a predominant HA-SA clone in China^18^. The ST398 isolates were significantly more virulent in the skin infection model compared with the ST239 isolates, inasmuch as the skin lesions of mice infected with ST398 isolates were significantly larger (Fig. 1B,C) and concentrations of the inflammatory cytokine TNF-α in abscess tissue significantly higher (Fig. 1D) than in mice infected with ST239 isolates.

In a previous study, in which we compared the genetic composition of ST398 versus ST239 and ST5 isolates, we only found minor differences in the presence of virulence genes^19^. Genes for the surface proteins SdrC and SdrE and those encoding PVL were significantly more prevalent, while a series of exotoxin genes were less prevalent in ST398. However, as mentioned above for PVL, these were not sufficiently widespread among ST398 isolates to explain for an overall difference in virulence of the lineage.

### CA-SA ST398 isolates show high expression of genes belonging to ESS

**Figure 2:**
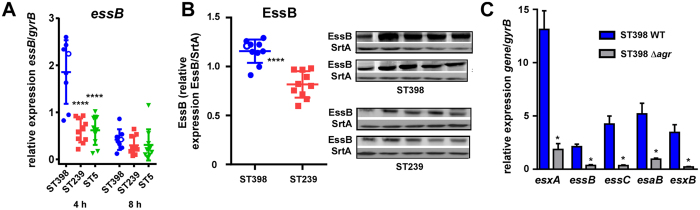
Toxin and Agr expression in ST398 and HA-SA isolates, and Agr regulation of ESS. (**A**) Quantitative RT-PCR analysis of *essB* gene expression in randomly selected clinical CA-SA ST398, HA-SA ST239, and HA-SA ST5 isolates, at 4 and 8 h of *in-vitro* growth. ****p < 0.0001 (1-way ANOVA with Dunnett’s post-test versus ST398). (**B**) Production of EssB, Western blot with EssB-specific antibodies. Production of constitutively expressed sortase A (SrtA), measured with specific anti-SrtA antibodies, was used for normalization. Densitometric values of the Western blot signals shown at the right were determined and compared. ****p < 0.0001 (Mann-Whitney). (**C**) Control of ESS gene expression by Agr, qRT-PCR. *p < 0.05; (Mann-Whitney). Four biological replicates per group were analyzed. The empty circles in (**A**,**B**) represent the data corresponding to the isolate selected for genetic deletion.

Increased expression of Agr-controlled toxins, in particular α-toxin, has recently been made responsible for the high virulence of ST398 isolates that caused fatal pneumonia^12^. In our previous study^19^, we showed that Agr (as measured by expression of RNAIII), α-toxin, and phenol-soluble modulins (PSMs) α also showed increased expression in our isolates. In contrast, the non-Agr-regulated exopolysaccharide biosynthesis locus *ica*^20^,^21^ did not show differential expression, and several of the often Agr-down-regulated surface protein genes showed the expected opposite mode of expression^19^. Here, we analyzed expression of genes belonging to ESS and found that also several of those (*essA*, *essB*, *essC*, *esxA*) showed significantly increased expression as compared to ST239 isolates (Fig. 2A, Supplementary Fig. S1). We confirmed increased expression of the protein EssB, which is an essential membrane-located component of ESS^22^, by Western blotting using antiserum developed against a purified glutathion-S-transferase (GST)-EssB fusion protein (Fig. 2B). These results demonstrated enhanced expression of several key ESS genes and proteins in ST398 clinical isolates and suggested that ESS is Agr-regulated in ST398. To directly verify that hypothesis, we constructed an *agr* mutant of a ST398 isolate and measured expression of ESS genes, which confirmed that ESS is under Agr control in that lineage (Fig. 2C).

### ESS of ST398 has a significant impact on acute virulence in experimental blood and skin infection

**Figure 3:**
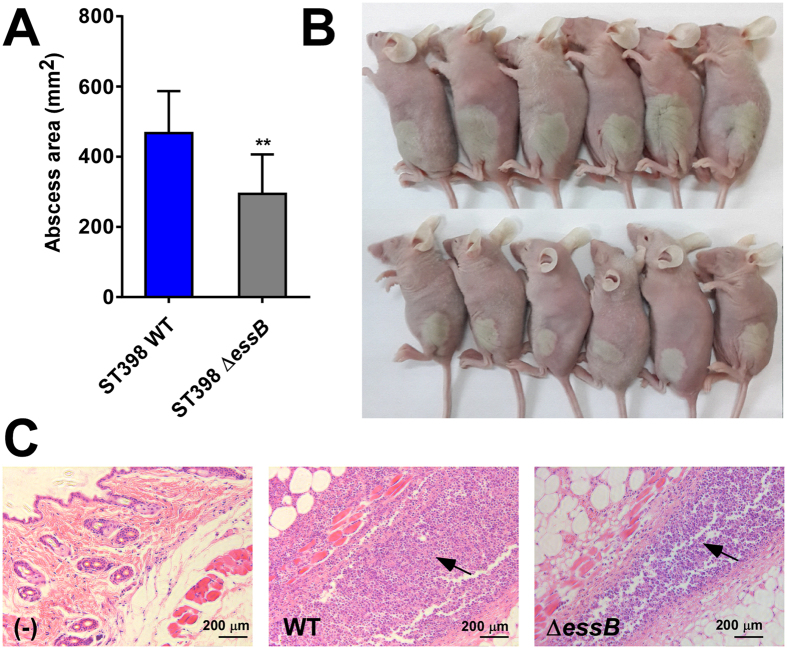
Role of ESS in CA-SA ST398 skin infection. Mouse abscess model with *essB* deletion mutant versus isogenic wild-type representative ST398 isolate. Number of mice used: n = 11/group. (**A**) Abscess areas on day 2 after infection. **p < 0.01 (unpaired t-test). (**B**) Representative abscesses on day 2 after infection. (**C**) H&E staining of abscess skin tissue harvested on day 4. (–), control without challenge. Note considerable infiltration of inflammatory cells and damage of the subcutaneous structure in the wild-type and *essB* mutant samples, noticeably stronger in the wild-type sample (arrows).

**Figure 4:**
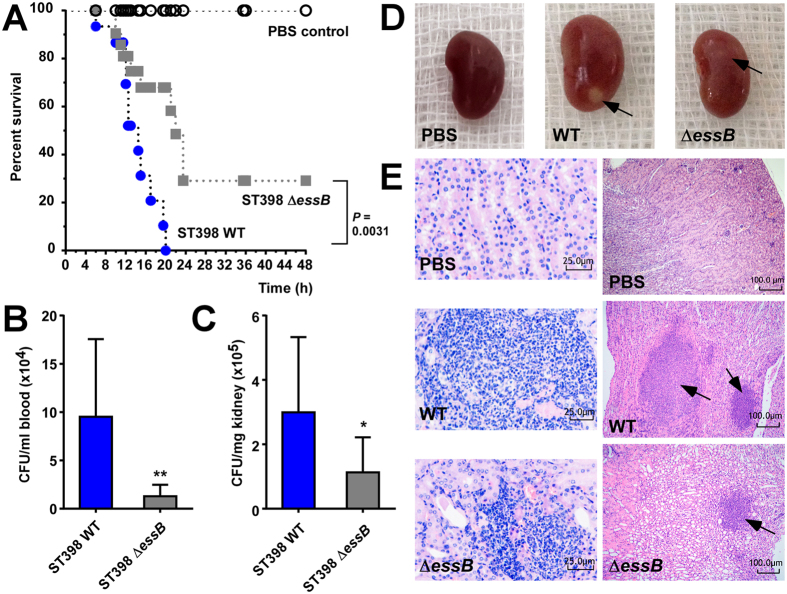
Role of ESS in CA-SA ST398 bacteremia. Mouse bacteremia model with *essB* deletion mutant versus isogenic wild-type representative ST398 isolate. (**A**) Survival analysis. Mice were injected with 10^9^ CFU live *S. aureus* or PBS. Survival curves were compared using a log-rank (Mantel-Cox) test. Number of mice used: n = 11 (wild-type); n = 10 (*essB* mutant and PBS). (**B-D**) Mice (n = 10/group) were infected with 10^8^ CFU live *S. aureus* and euthanized 4 days after injection. CFU in blood (**B**) and kidneys (**C**) were assessed by plating on TSB agar. *p < 0.05; **p < 0.01 (unpaired t-tests). For CFU determination, n = 9/group (two mice had to be excluded due to blood coagulation and resulting difficulties in CFU determination). (**D,E**) Photographs of kidneys (**D**) and corresponding H&E stained sections harvested from mice 4 days after injection (**E**). Note formation of macroscopic abscesses in kidneys of mice infected with wild-type and *essB* mutant strains (**D)** (arrows), with the abscess being bigger in the wild-type-infected kidney. In (**E**), note considerable infiltration of inflammatory cells in the wild-type and *essB* mutant samples, noticeably stronger in the wild-type sample (arrows).

Our results suggest that the observed rise in ST398 infections is due to enhanced virulence of the ST398 isolates and associated with enhanced expression of determinants of acute virulence. Based on our expression analyses, we hypothesized that ESS may represent a previously underestimated component contributing to acute virulence of emerging CA-SA strains such as ST398. To evaluate that hypothesis, we constructed an isogenic gene deletion mutant in *essB*, an essential component of ESS, in a representative (based on our gene expression and animal virulence data, Figs 1 and 2) ST398 isolate. We then compared the virulence potential of that mutant to that of the isogenic wild-type strain in mouse models of skin infection and bacteremia. In the skin infection model, the wild-type strain caused significantly larger abscesses than the *essB* mutant strain (Fig. 3A,B). Furthermore, histological examination showed increased infiltration of neutrophils and skin tissue destruction in the mice infected with the wild-type strain (Fig. 3C). In a high-dose (10^9^ CFU) bacteremia model, the mice infected with the wild-type strain died significantly faster than those infected with the *essB* mutant (Fig. 4A). In a low-dose (10^8^ CFU) bacteremia model, CFU counts in the blood and kidneys of mice 4 days after infection were higher in the mice infected with the wild-type strain than in those infected with the *essB* mutant strain (Fig. 4B,C). Furthermore, the kidneys of mice infected with the wild-type strain showed increased abscess formation (Fig. 4D). Moreover, histological examination showed more extensive inflammation accompanied by an increased number of infiltrating inflammatory cells in the kidneys of those mice (Fig. 4E). These results showed that ESS is a significant contributor to acute virulence in ST398.

### ST398 ESS is responsible for the secretion of EsxA and EsxB

**Figure 5:**
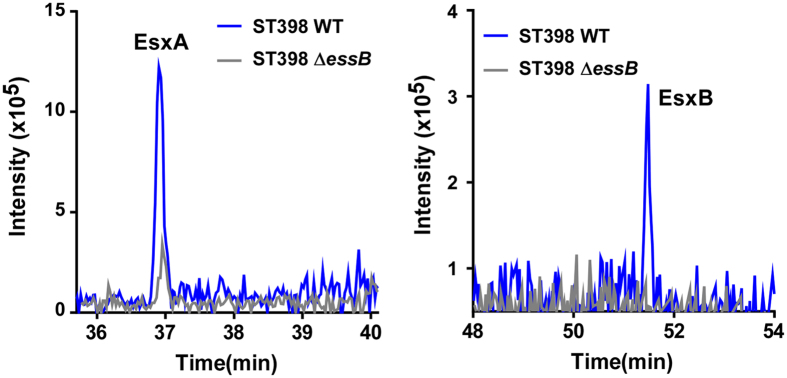
ESS controls the secretion of EsxA and EsxB proteins in CA-SA ST398. Equal amounts of secreted proteins from RJ-ST398 wild-type and *essB* deletion strains were separated by 12% SDS-PAGE and in-gel digested by trypsin before LC-MS/MS analysis. Peaks of peptides derived from EsxA (AQGEIAANWEGQAFSR) and EsxB (LQDAVNSAQTIIEDFNSEK) are shown.

To gain insight into how the ESS of ST398 contributes to virulence, we first analyzed whether it is involved in the secretion of EsxA and EsxB, which have been previously described as virulence factors that are secreted by ESS in other *S. aureus* strains^13^. According to LC-MS/MS analysis of ST398 wild-type versus *essB* deletion mutant culture filtrates, EsxA and EsxB are secreted in ST398 in an ESS-dependent fashion (Fig. 5). Then, to rule out that the impact of ESS on ST398 virulence is mediated by a regulatory influence on major toxins and toxin regulators previously associated with ST398 virulence, we measured expression of *hla*, *psm*α, and Agr in the *essB* mutant versus the wild-type ST398 isolate, showing that ESS of ST398 does not have such a regulatory effect (Supplementary Fig. S2). Thus, our results indicated that the impact of ESS on virulence is mediated predominantly by virulence factors whose secretion depends on ESS, such as EsxA and EsxB.

### ESS of ST398 promotes resistance to elimination by human neutrophils

**Figure 6:**
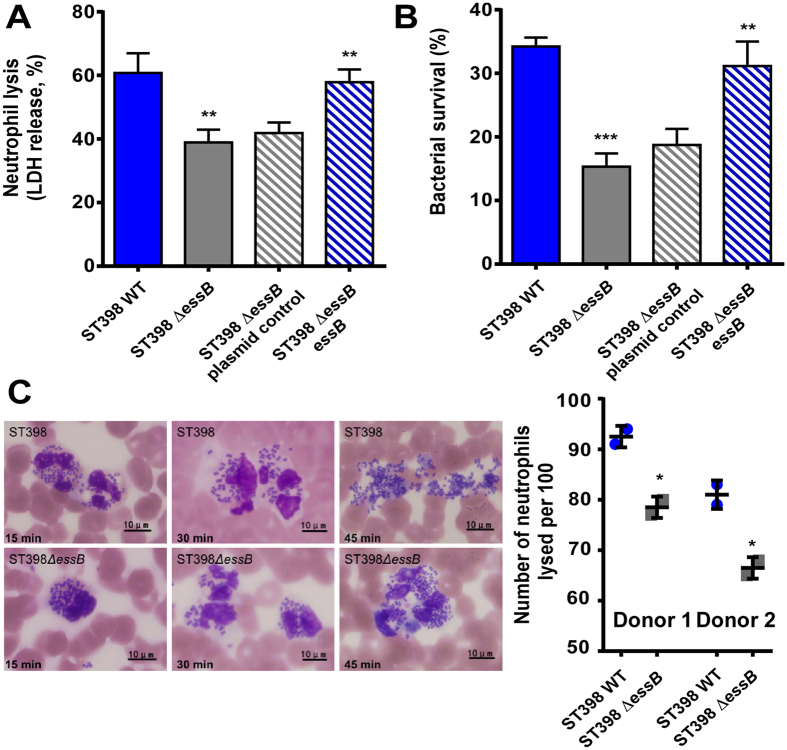
ESS of CA-SA ST398 has an important role in evasion of elimination by human neutrophils. Human neutrophils were isolated from heparinized venous blood of healthy individuals. Bacteria from mid-logarithmic growth phase were washed and incubated with human neutrophils at a 10:1 ratio. (**A**) Neutrophil lysis was determined by measuring release of LDH. (**B**) Bacterial survival was measured by CFU plating. **p < 0.01; ***p < 0.001 (unpaired t-tests, deletion mutant versus wild-type, and complemented mutant versus deletion mutant harboring control plasmid). (**C**) Microscopic evaluation of lysis after phagocytosis. Blood smears were obtained at different time points of incubation of bacteria with neutrophils, and ingestion and lysis were analyzed by light microscopy using a modified Wright-Giemsa stain. The number of lysed among all (=100) neutrophils that had ingested bacteria is shown on the right for two different donors. *p < 0.01 (unpaired t-tests).

The mechanism by which ESS contributes to virulence has remained mostly unclear. In a previous study, ESS was found to impact colonization^14^, during which staphylococci are believed to grow in a biofilm mode of growth^23^. We therefore measured the impact of ST398 ESS on biofilm formation. However, no impact of ST398 ESS on biofilm formation was found (Supplementary Fig. S3). Recently, EsxA and EsxB were reported to delay apoptosis and contribute to release of *S. aureus* from epithelial cells^16^. These results prompted us to investigate whether ESS impacts *S. aureus* interaction with professional phagocytes, in particular neutrophils, which represent the first line of defense against an *S. aureus* infection. An interaction with this key part of innate host defense would explain the impact of ESS on acute virulence that we observed in the animal infection models. Thus, we analyzed the degree of bacterial survival and lysis of neutrophils upon incubation of wild-type versus *essB* mutant bacteria with human neutrophils. Bacterial survival was significantly higher and lysis of neutrophils significantly greater with wild-type as compared to *essB* mutant bacteria (Fig. 6A,B). In contrast to PSMs, which are lytic to a variety of cells including neutrophils^24^, the cytolytic effect of ESS was specific, as the ESS did not influence lysis of erythrocytes (Supplementary Fig. S4). Furthermore, microscopic analysis indicated that ESS contributes to lysis after ingestion (Fig. 6C). These results revealed that ESS is an important factor determining interaction of ST398 with neutrophils as main effectors of innate host defense.

## Discussion

The recent decline in *S. aureus* hospital infections, which is likely due to increased hygienic measures, unfortunately is accompanied by an ongoing global surge of CA-SA infections^2^,^6^. CA-SA infections are commonly caused by clones that are entirely different from those encountered in hospitals^6^. While the ST398 lineage has only sporadically and to a minor extent been known to cause such infections, our present study reveals that it has grown to a considerable problem in China, representing the source for about one third of CA-SA infections in Shanghai. Notably, our data also indicate that the ST398 cases are not related to contact with livestock, which is in accordance with data from New York and Brazil^11^,^12^.

The molecular underpinnings of what underlies the epidemiological success of CA-SA have remained poorly understood. The methicillin-resistant CA-SA clone USA300, the main source of CA-MRSA infections in the United States, has received much attention^6^,^7^. In that clone, epidemiological success has been linked to the acquisition of factors encoded on mobile genetic elements, such as the Panton-Valentine leukocidin (PVL), the arginine catabolic mobile element (ACME), and a comparatively small methicillin resistance element, SCCmec IV (or V)^7^. With PVL only present in a minority of CA-SA ST398 isolates in our study, this leukocidin cannot be made responsible for the surge in ST398 CA-SA infections that we observed in Shanghai. Rather, our results showing increased expression in ST398 of genome-encoded toxins such as PSMs and α-toxin, and increased expression of the Agr global virulence regulator, which controls many of the corresponding genes, is in accordance with the hypothesis that enhanced production of those toxins has an important role in the virulence potential of CA-SA^19^.

The present study focused on ESS and adds ESS to the Agr-controlled factors that increase pathogenic success and acute virulence potential in *S. aureus*. Furthermore, our data indicate that increased expression of ESS may have contributed to the surge in ST398 infections that we observed. Whether ESS has a similarly important role in other CA-SA strains remains to be investigated. Of note, our findings that ESS is Agr-regulated and ST398 isolates show exceptionally high expression of Agr and other Agr-controlled toxins help to explain reports on strain dependency, such as the reported lack of contribution of ESS to virulence in the functionally Agr-negative strain SA113^14^.

Finally, our data give important insight into how ESS and the ESS-dependent secreted virulence determinants EsxA and EsXB promote acute virulence. Namely, the observed impact of ESS on neutrophil killing provides a valid explanation of that influence, inasmuch as neutrophils represent the cornerstone of human innate defenses against *S. aureus* infection.

In conclusion, our study reveals ST398 as an epidemiologically important CA-SA lineage in China. Furthermore, it provides evidence for a previously unrecognized role of ESS in acute virulence of CA-SA strains and links that phenomenon to a role of ESS in resistance to elimination by neutrophils as a pivotal component of innate immunity. In general, our findings support the notion of a key contribution of enhanced production of toxins to the evolution of virulent CA-SA strains.

## Materials and Methods

### Ethics statement

Heparinized venous blood was obtained from healthy human volunteers in accordance with a protocol approved by the ethics committee of Renji Hospital, School of Medicine, Shanghai Jiao Tong University, Shanghai (protocol RJ-H-2014-0213). All individuals gave informed consent prior to donating blood. All animal experiments were performed following the Guide for the Care and Use of Laboratory Animals of the Chinese Association for Laboratory Animal Sciences (CALAS) and approved by the ethics committee of Renji Hospital, School of Medicine, Shanghai Jiao Tong University, Shanghai (protocol RJ-M-2014-0109).

### Bacterial strains, plasmids, oligonucleotides, and growth conditions

Laboratory-based surveillance for the molecular characterization of clinical *S. aureus* infection was performed in one comprehensive teaching hospital for adult patients in Shanghai, China (Renji Hospital affiliated to Shanghai Jiao Tong University) and one teaching hospital for pediatric patients (Shanghai Children’s Medical Center affiliated to Shanghai Jiaotong University). Bacteria were identified as staphylococci by classic microbiological methods: Gram’s stain, catalase and coagulase activity on rabbit plasma. *S. aureus* strains were further categorized by biochemical characterization using the Api-Staph test (BioMérieux, France). CA-SA was defined as an isolate that was obtained either from an outpatient or from an inpatient ≤24 h after hospital admission and without the patient having any of the following risk factors: contact with the hospital environment in the preceding 6 months, residence in a long-term care facility in the preceding 12 months, *S. aureus* infection in the preceding 12 months, presence of a central vascular catheter at the time of infection. HA-SA was defined as an isolate that was obtained from an inpatient >24 h after hospital admission, or from an outpatient or inpatient ≤24 h after hospital admission, but with the risk factors listed above. Laboratory strains and plasmids used in this study are listed in Table 2. *Escherichia coli* was routinely grown in Luria-Bertani medium, *S. aureus* was grown in tryptic soy broth (TSB) (Oxoid) with 0.25% glucose or agar plates at 37 °C. Antibiotics were used at the following concentrations: ampicillin, 100 μg/ml; chloramphenicol, 10 μg/ml. All oligonucleotides used in the present study are listed in Table 3.

### Molecular typing

Molecular typing was performed using multi-locus sequence typing (MLST) as previously described^25^. PCR amplicons of seven *S. aureus* housekeeping genes (*arcC*, *aroE*, *glpF*, *gmk*, *pta*, *tpi*, and *yqiL)* were obtained from chromosomal DNA. The sequences of the PCR products were compared with the existing sequences available at the MLST website (http://www.mlst.net).

### Allelic gene replacement by homologous recombination and genetic complementation

To delete the *essB* gene from the genome of *S. aureus* ST398, a representative clinical isolate (RJ-ST398) was chosen, which was recovered from the abscess of a patient with skin infection. The homologous recombination procedure was performed as described^26^, using plasmid pKOR1. DNA fragments for upstream and downstream sequences of *essB* were PCR amplified from chromosomal DNA of RJ-ST398, and overlap PCR was used to obtain a fused PCR product. This product was cloned into pKOR1 using clonase reaction and *attB* sites, yielding plasmid pKOR1Δ*essB*. The resulting plasmid was transferred via electroporation first to *S. aureus* RN4220 and then RJ-ST398. The allelic replacement procedure was performed as described^27^. Proper integration was verified by analytical PCR and sequencing of the genomic DNA at the borders of the PCR-derived regions. For genetic complementation, the *essB* gene was amplified by PCR with primers EssB-SmaI-F and EssB-BamHI-R. The *essB* complementation plasmid was generated by cloning the *essB* gene in vector pOS1. Growth of the *essB* deletion and complemented mutants was comparable to that of the wild-type strain (Supplementary Fig. S5). Deletion of the *agr* system in RJ-ST398 was performed in principle as described for *essB*.

### Quantitative reverse-transcription (RT) PCR

Complementary DNA was synthesized from total RNA using the QuantiTect reverse transcription system (Qiagen) according to the manufacturer’s instructions. Oligonucleotide primers were designed using Primer Express. The resulting complementary DNA and negative control samples were amplified using the QuantiTect SYBR green PCR kit (Qiagen). Reactions were performed in a MicroAmp Optical 96-well reaction plate using a 7500 Sequence Detector (Applied Biosystems). Standard curves were determined for each gene, using purified chromosomal DNA at concentrations of 0.005–50 ng/ml. All quantitative reverse-transcription polymerase chain reaction (qRT-PCR) experiments were performed in duplicate, unless noted otherwise, with *gyrB* as control.

### Construction and purification of GST-Tag fusion protein and antiserum

The *essB* gene was cloned and overexpressed as glutathione-S-transferase (GST) fusion. The PCR products were purified and digested with BamHI and EcoRI. The genes were cloned into the GST gene fusion vector pGEX-4T-1 (Amersham Biosciences). The recombinant vector pGEX-*essB* was maintained in *E. coli* strain BL21 (Amersham Biosciences) for overexpression. Cultures were grown at 30 °C with aeration to an OD_600_ of 1.0. IPTG was subsequently added to a final concentration of 0.5 mM, and the culture was incubated with aeration for an additional 5 h. Then the cells were harvested. The fusion protein was purified using a GST-Tag purification Kit (Chemicon) according to the manufacturer’s instructions. The purified fusion protein was concentrated in Centriprep-10 concentrators (Amicon) and dialysed against 10 mM sodium phosphate buffer (pH 7.5) using PD-10 desalting columns (Amersham Biosciences). The size of the GST fusion protein was confirmed by SDS–PAGE. The purified protein was used to immunize rabbits by Xiangtai Biotech Ltd (Changzhou, China) according to their standard immunization program.

### Western blot analysis

Overnight cultures were diluted 1:100 into 50 ml of TSB and incubated at 37 °C with shaking at 200 rpm until grown to an OD_600 nm_ of 1.5–2.0 (mid-logarithmic growth phase). 500 μl bacterial culture was then collected, cells were harvested and resuspended in 50 μl TE buffer (10 mM Tris-HCl, 1mM EDTA, pH 8.0), lysostaphin (100 μg/ml) was added, and cells were incubated for 30 min at 37 °C. Samples were mixed with protein loading buffer and boiled for 10 min. Equivalent amounts of proteins were separated by SDS-PAGE and transferred onto nitrocellulose membranes (Invitrogen). After blocking, the membranes were incubated with EssB antiserum at 4 °C overnight and then incubated with horseradish peroxidase-conjugated secondary antibody at room temperature for 1 h. Images of Western blots were acquired using a Tanon-5200 system. Intensities of reactions with SrtA antibody (kindly provided by Taeok Bae, Indiana University School of Medicine-Northwest, Gary, Indiana) were used as controls to determine loading amounts.

### Analysis of hemolytic capacity

Strains were grown to early stationary growth phase (4 h), and equal amounts (2 μl) of cells were spotted on sheep blood agar plates and incubated at 37 °C for 24 h.

### Semiquantitative biofilm assay

Semiquantitative biofilm assays were performed as described elsewhere^27^. Subsequently, cells were fixed by Bouin’s fixative. The fixative was removed after 1 h and wells were washed with PBS. Organisms in the wells were then stained with crystal violet, and the floating stain was washed off with slow- running water. After drying, the stained biofilm was read with a MicroELISA autoreader (BioRad) at 570 nm (crystal violet stain).

### Mass spectrometric analysis

Filtrates of cultures grown to an OD_600 nm_ of 5.0 were precipitated with 10% trichloroacetic acid (TCA), washed with ice-cold acetone, and then resuspended in 50 mM Tris-HCl pH 8.0, 4% sodium dodecylsulfate (SDS). Samples were mixed with protein loading buffer and boiled for 10 min. 12% SDS-PAGE was used to pre-fractionate the secreted proteins. Protein samples were subjected to in-gel digestion. Finally, the extracted peptides were vacuum-dried prior to LC-MS/MS analysis on a nanoflow liquid chromatography instrument (EASY-nLC 1000, Thermo Scientific) coupled to an ion trap mass spectrometer (LTQ Velos Pro. Thermo Scientific) in data-dependent mode. Detailed LC-MS/MS settings have been described elsewhere^28^.

### Neutrophil lysis and bacterial survival assays

Neutrophil lysis by whole bacteria was measured using a lactate dehydrogenase (LDH) cytotoxicity detection kit according to the manufacturer’s protocol (Roche) as described elsewhere^18^. Bacteria were grown to mid-logarithmic growth phase (4 h). Neutrophils and bacteria were used at a 1:10 ratio (MOI 10) and incubated for 3 h before detection of LDH activity. Samples were diluted 1:50 for the assays. To determine survival rates, 100 μl of the culture above was plated on TSA containing 5% sheep blood for *S. aureus* CFU detection.

For microscopic evaluation of neutrophil killing after phagocytosis, overnight cultures were diluted 1:100 into 50 ml of TSB and incubated at 37 °C with shaking at 200 rpm to late logarithmic growth phase (4 h), washed, and resuspended in sterile phosphate-buffered saline (PBS) at 10^7^ CFU/μl. *S. aureus* cells were mixed with heparinized venous blood at a ratio of blood/bacterial solution of 1:10 (v/v) and incubated at 37 °C. Blood smears were prepared after the indicated times of incubation and cells were stained with a modified Wright-Giemsa stain. The number of neutrophils lysed by *S. aureus* among the total number of neutrophils that had ingested *S. aureus* (n = 100 were analyzed) was calculated by microscopic examination.

### Mouse bacteremia and skin abscess models

BALB/c female mice were used for the bacteremia model. Outbred, immunocompetent hairless female mice were used for the abscess model. All mice were between 4 and 6 weeks of age at the time of use. *S. aureus* strains were grown to mid-logarithmic phase, washed once with sterile phosphate buffered saline (PBS), then resuspended in PBS. For the bacteremia model, the BALB/c female mice were infected with live *S. aureus*. Mice of group I were injected with 100 μl PBS containing 10^9^ CFU and used for survival analysis; mice of group II were injected with 10^8^ CFU. Control animals received PBS only. After inoculation, mouse health and disease advancement were monitored every day, and mice were euthanized immediately if they showed signs of respiratory distress, mobility loss, or inability to eat and drink. All surviving animals in group I were euthanized at 48 h. The surviving animals in group II were euthanized 4 days after injection. In group II, *S. aureus* CFU in blood were assessed by plating 10 μl blood on TSB agar. Serum samples were used for TNF-α detection. Kidneys were excised, washed with saline, and one kidney was fixed in 4% formalin (Sigma). Paraffin embedding and hematoxylin & eosin (H&E) staining were performed as previously described^18^. The other kidney was homogenized in 0.5 ml of TSB, the homogenized kidney tissue diluted and plated on TSB agar for determination of CFU.

For the abscess model, mice were anesthetized with isoflurane and inoculated with 50 μl PBS containing 10^7^ live *S. aureus* or saline alone in the left flank by subcutaneous injection. Animals were examined at 24-h intervals for a total of 4 d. Abscess length (L) and width (W) values were measured to calculate the area of abscesses with the formula area (A) = π (L × W)/2. All animals were euthanized 4 days after injection. Abscess material samples of 0.05 g were homogenized in 0.5 ml PBS for the measurement of the cytokine TNF-α (Bio-Plex Pro Mouse Cytokine; Bio-Rad Laboratories).

### Statistics

Statistical analysis was performed using Graph-Pad Prism, version 6.02. Error bars in all graphs show the standard deviation (±SD).

## Additional Information

**How to cite this article**: Wang, Y. *et al*. Role of the ESAT-6 secretion system in virulence of the emerging community-associated *Staphylococcus aureus* lineage ST398. *Sci. Rep*. **6**, 25163; doi: 10.1038/srep25163 (2016).

## Supplementary Material

Supplementary Information

## Figures and Tables

**Table 1:** Characteristics of CA-SA ST398 isolates from adult and pediatric patients.

	Isolation of CA-SA ST398			Isolation of HA-SA ST398		Spa type			Infection type		
	Year	No./total CA-SA	%	No./total HA-SA	%		No./total CA-SA	%		No./total CA-SA	%
Adult	2005–2010	12/56	21.4	3/446	0.7	t034 t571 others	25/69 24/69 20/69	36.2 34.8 29.0	SSTIs respiratory bacteremia others	32/69 24/69 5/69 8/69	46.4 34.8 7.3 11.6
	2011	15/78	19.2	5/522	1.0						
	2012	15/65	23.1	4/413	1.0						
	2014	27/81	33.3	8/389	2.1						
	total	69/280	24.6	20/1770	1.1						
Children	2012	14/57	24.6	4/87	4.6	t034 t571 others	8/14 4/14 2/14	57.1 28.6 14.3	SSTIs respiratory others	8/14 4/14 2/14	57.1 28.6 14.3

**Table 2:** Bacterial strains and plasmids used in this study.

Strains/plasmids	Relevant genotype and property	Source/reference
*S. aureus*		
RN4220	derived from NCTC8325-4;r-m+	29
RJ-ST398	CA-SA clinical isolate	This study
RJ-ST398Δ*essB*	RJ-ST398 *essB* mutant	This study
RJ-ST398Δ*essB* (pOS1)	RJ-ST398 *essB* mutant with pOS1	This study
RJ-ST398Δ*essB* (pOS1*essB*)	398 *essB* mutant with pOS1*essB*	This study
RJ-ST398Δ*agr*	RJ-ST398 *agr* mutant	This study
*E. coli*		
DH5α	*endA*1 *recA*1 *gyrA*96 *thi-1 hsdR17*(rK- mK+) *relA*1 *supE*44 (*lacZYA-argF*)U169 F-80d*lacZ*M15 *deoR phoA*	Invitrogen
BL21	*E. coli B F- dcm ompT hsdS*(rB- mB-) gal For recombinant protein production	Amersham Biosciences
*Plasmids*		
pKOR1	cmR and ampR, temperature-sensitive vector for allelic replacement via lambda recombination and *ccdB* selection	26
pKOR1Δ*essB*	Vector for allelic replacement of *essB* in *S. aureus*	This study
pKOR1Δ*agr*	Vector for allelic replacement of *agr* in *S. aureus*	This study
pOS1	*E. coli/Staphylococcus* shuttle cloning plasmid, *cm*R, *amp*R	30
pOS1*essB*	pOS1 with insertion of *essB* gene	This study
pGEX-4T-1	ampR,for recombinant GST fusion protein production	Amersham Biosciences
pGEX-*essB*	pGEX-4T-1containing the *essB* gene of *S. aureus* ST398	This study

**Table 3:** Oligonucleotides used in this study.

Oligonucleotide	Sequence
*Oligonucleotides for isogenic deletion mutants*	
essB-att1	GGGGACAAGTTTGTACAAAAAAGCAGGCTGATGGCGACAGACAAAACCAAGC
essB-rev1	CTATTTTTCCTCCTATAGTAACTTC
essB-rev2	GTTACTATAGGAGGAAAAATAGGTATAGGACTGAGGCAAAGACAATGC
essB-att2	GGGGACCACTTTGTACAAGAAAGCTGGGTGTGCCCATTTCAATGTTTCAAC
agr-att1	GGGGACAAGTTTGTACAAAAAAGCAGGCTACCCTTTCAATTGTCTGACG
agr -rev1	GATGAATAATTAATTACTTTCATTGTAAA
agr-rev2	AGTAATTAATTATTCATCACTTACCTATTTAACGTTTGTCTACA
agr-att2	GGGGACCACTTTGTACAAGAAAGCTGGGTGGGATGCCTTTATTGGTG
*Oligonucleotides for genetic complementation*	
EssB-Sma1-F	GAGCCCGGGAGATGGTTAAAAATCATAACCCTAAAAATG
EssB-BamH1-R	GAGGGATCCCTATTTTTTTCTTTCAGCTTCTT
*Oligonucleotides for qRT- PCR*	
gyrB-F	CAAATGATCACAGCATTTGGTACAG
gyrB-R	CGGCATCAGTCATAATGACGAT
essB-F	TTTTCACAAGAGATGGATTACCAATT
essB-R	TTCTGACACCGGTAATGGATCA
esxA-F	CGTGCACAAGGTGAAATTG
esxA-R	CGAAACGGCTGAAAGCTT
essA-F	AAAAGCCCCTATATTCAAAACAA
essA-R	GCCCCTACAGACATCAAAATG
essC-F	TGCGTGCCTTAACATCAATC
essC-R	TAACGTCATGCTCTCCGAATAA
esaA-F	GTCAAGATGAACCAAATGATAAGAA
esaA-R	CCATCAAGGTTATGATTCAATGTT
esaB-F	CAAATTAAAGTGATGACGAAAGGT
esaB-R	CTCCATCAGCGATTTGATAATC
*Oligonucleotides for construction and purification of GST-tagged fusion protein*	
ESSB-BamH1-F	GAGGGATCCATGGTTAAAAATCATAACCCTAAAAATG
ESSB-EcoR1-R	GAGGAATTCCTATTTTTTTCTTTCAGCTTCTT
