# Supplementary material for: Role of the ESAT-6 secretion system in virulence of the emerging community-associated Staphylococcus aureus lineage ST398
Source: Sci Rep. 2016 Apr 26;6:25163. doi: 10.1038/srep25163 (PMC4844983; doi:10.1038/srep25163)
Supplement: Supplementary Information [file srep25163-s1.pdf]

**Role of the ESAT-6 secretion system in virulence of the emerging community-associated *Staphylococcus aureus* lineage ST398**

Yanan Wang, Mo Hu, Qian Liu, Juanxiu Qin, Yingxin Dai, Lei He, Tianming Li, Bing Zheng, Fan Zhou, Kaiwen Yu, Jingyuan Fang, Xiaoyun Liu, Michael Otto, Min Li

## Supplementary Information

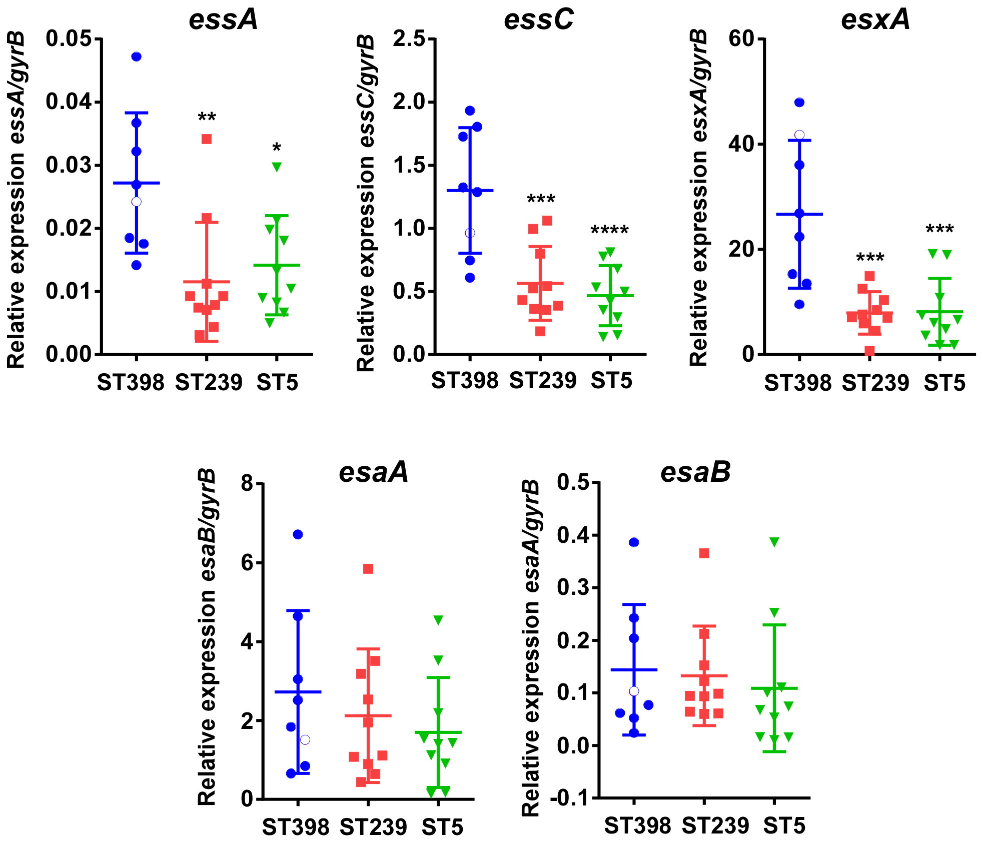


**Fig. S1. Expression of ESS genes in CA-SA ST398 versus HA-SA randomly selected isolates.** Data are from culture grown for 4 h. The empty circles represent the data corresponding to the isolate selected for genetic deletion. *, p<0.05; **, p<0.01; ***, p<0.001 ****, p<0.0001 (1-way ANOVA, Dunnett's post test vs. ST398).

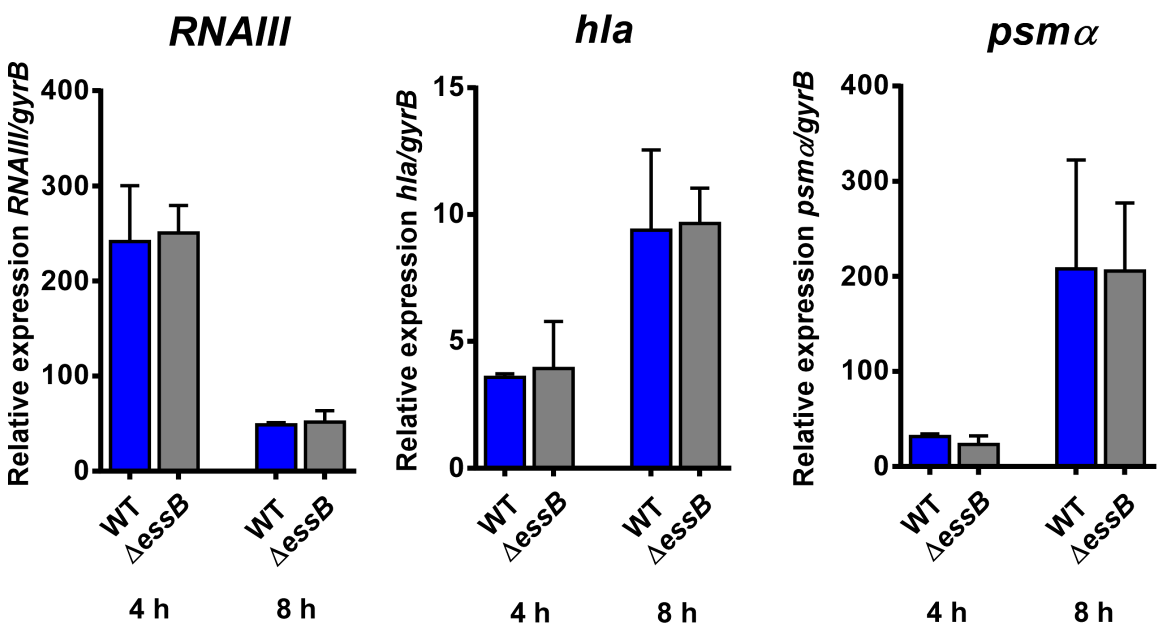


**Fig. S2. Expression of RNAIII (Agr), *hla*, and *psm*α in ST398 wild-type versus *essB* deletion mutant strains at 4 and 8 h of growth.**

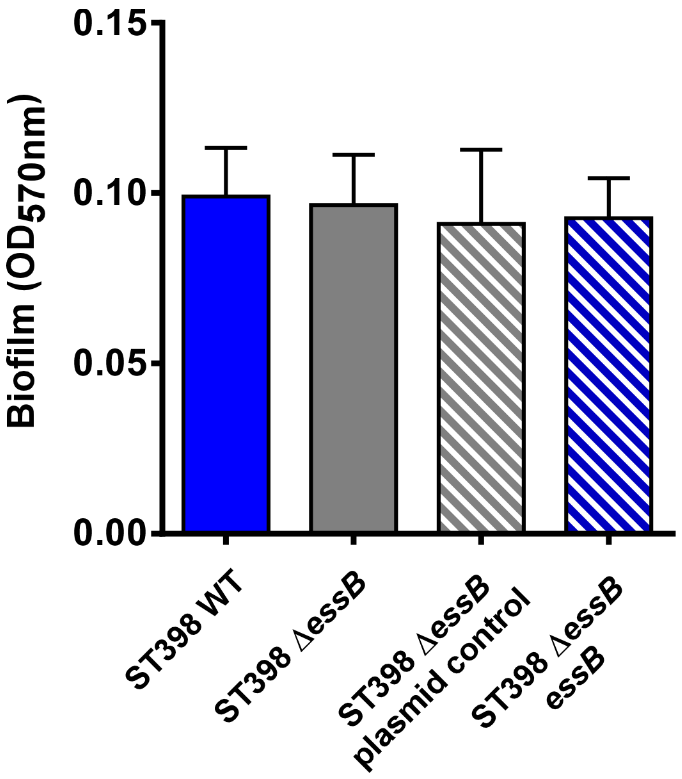


**Fig. S3. Biofilm formation by ST398 wild-type, *essB* deletion mutant, complemented mutant, and control strains.**

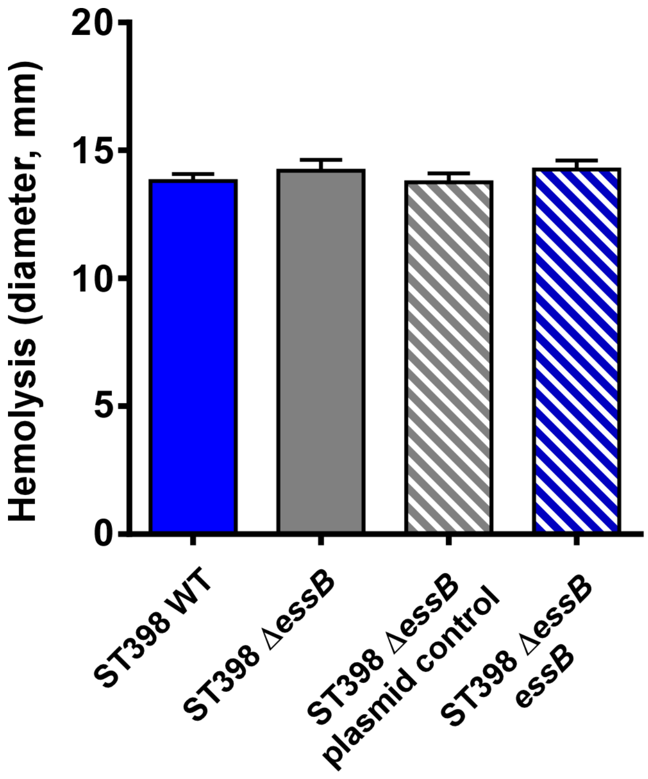


**Fig. S4. Hemolysis by ST398 wild-type, *essB* deletion mutant, complemented mutant, and control strains.**

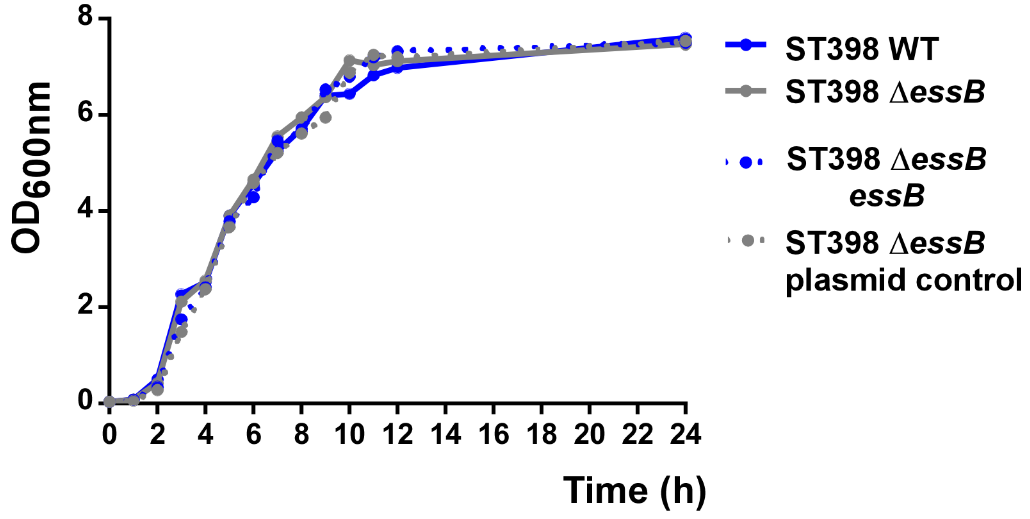


**Fig. S5. Growth curves of strains used in this study.**
